# Impact of Gut Microbiota on Host Aggression: Potential Applications for Therapeutic Interventions Early in Development

**DOI:** 10.3390/microorganisms11041008

**Published:** 2023-04-12

**Authors:** Katsunaka Mikami, Natsuru Watanabe, Takumi Tochio, Keitaro Kimoto, Fumiaki Akama, Kenji Yamamoto

**Affiliations:** 1 Department of Psychiatry, Tokai University School of Medicine, Isehara 259-1193, Kanagawa, Japan; 2 Department of Gastroenterology and Hepatology, Fujita Health University, Toyoake 470-1192, Aichi, Japan

**Keywords:** aggression, early development, germ-free mice, gut microbiota, microbiota–gut–brain axis, prevention, therapeutic intervention, vertical transmission

## Abstract

Aggression in the animal kingdom is a necessary component of life; however, certain forms of aggression, especially in humans, are pathological behaviors that are detrimental to society. Animal models have been used to study a number of factors, including brain morphology, neuropeptides, alcohol consumption, and early life circumstances, to unravel the mechanisms underlying aggression. These animal models have shown validity as experimental models. Moreover, recent studies using mouse, dog, hamster, and drosophila models have indicated that aggression may be affected by the “microbiota–gut–brain axis.” Disturbing the gut microbiota of pregnant animals increases aggression in their offspring. In addition, behavioral analyses using germ-free mice have shown that manipulating the intestinal microbiota during early development suppresses aggression. These studies suggest that treating the host gut microbiota during early development is critical. However, few clinical studies have investigated gut-microbiota-targeted treatments with aggression as a primary endpoint. This review aims to clarify the effects of gut microbiota on aggression and discusses the therapeutic potential of regulating human aggression by intervening in gut microbiota.

## 1. Introduction

All animal species exhibit species-specific aggressive behavioral patterns, which are regarded as some of the earliest emotional behavioral patterns to have evolved [1]. In the animal kingdom, aggression is evolutionarily conserved for the purpose of protecting valuable resources, such as breeding partners, offspring, food, and territory, and for establishing and maintaining social status and hierarchy. Thus, aggression may be considered as a normal and necessary component of social behavior [1,2,3]. However, some forms of aggression, particularly in humans, are hostile, injurious, or destructive, and may be collective or individual [4]. Aggressive behavioral patterns that threaten lives are categorized under pathological behaviors associated with mental illness, and place a significant economic burden on society [3]. Notably, despite the costs involved, pervasiveness among psychiatric patients, and a general lack of treatments, human aggression has generated only a few studies compared with other affective behavioral patterns [3]. Thus, animal models are used to investigate the modulators as well as the causal mechanisms underlying pathological aggressive behavior in humans. Several mechanisms underlying aggressive behavior have been proposed. This review attempts to clarify the effects of gut microbiota on aggression considering animal models, and discusses the therapeutic potential of regulating human aggression by intervening in gut microbiota.

## 2. Animal Models of Aggression

Although the results of clinical studies are imperative, it is difficult to determine causal relationships when studying humans due to the diversity of factors influencing the outcome. Therefore, animal models are often used to analyze the mechanisms underlying aggression with a view to elucidating possible causal relationships. Various animal aggression models have been reported thus far [2].

Past winning experiences increase the probability of winning a subsequent contest, whereas past losing experiences decrease that probability, reflecting a modification in the expected fighting ability [5]. In many species, repetitively winning social conflict episodes increases the level of aggressiveness, as well as the probability of winning further aggressive encounters [2,5]. Winning an aggressive contest often results in a self-reinforcing or rewarding effect [2].

Investigating the association between brain morphology and aggression has revealed that reward regions, such as the nucleus accumbens and the lateral habenular nucleus of the brain, may play a functional role in reinforcing aggression [6]. Bioactive substances involved in brain functionally activate this neural network. Several bioactive substances, including monoamine dopamine (DA) [7], serotonin (5-hydroxytryptamine) [8,9], and the neuropeptides oxytocin [10] and vasopressin [11], are known to influence the magnitude of aggression. Animals with increased aggression exhibit 5-HT1A autoreceptor hypersensitivity and a reduced 5-HT reuptake function, reflecting a possible causal link in the cascade of neurochemical events leading to 5-HT deficiency that characterizes such violent animals [2]. Bioactive substances that influence aggression include glucocorticoid levels determined by the hypothalamic–pituitary–adrenal (HPA) axis. Administering glucocorticoids to brain centers increases aggression, resulting in positive feedback between the brain centers involved in regulating aggression and the stress response. Aggressive behavior increases glucocorticoid production even further, resulting in a vicious cycle [12].

Alcohol, which is considered the most potent psychoactive substance, has been shown to promote violent aggression and reduce behavioral control in a subset of human individuals [13]. A study examining the association between alcohol and aggression in rats suggested that the aggression-enhancing effects of alcohol are independent of baseline aggression levels [14,15]. Furthermore, alcohol may prolong aggressive outbursts by inhibiting their termination rather than altering the initiation of aggressive behavior [14]. A characteristic decrease in serotonin function in the frontal cortex has been associated with an increase in aggressive tendencies enhanced by alcohol [15].

Moreover, several animal models have suggested that experiencing adversity in childhood and stress may exert significant effects on social and aggressive behavior in adults. It has been shown that experiencing childhood adversity (e.g., emotional neglect, parental loss, and child abuse) can influence future aggression [16,17]. Rats subjected to social isolation from weaning onwards showed a dramatic increase in the proportion of attacks to the weaker parts of an opponent’s body (head, throat, and belly) and a shift from threats to real violence, suggesting a decrease in intentionality [18].

A range of factors, including brain morphology and brain function, neuropeptides, alcohol intake, and early life circumstances, have been investigated using a variety of animal models to determine the mechanisms underlying aggression. These animal models have shown validity as experimental models. Moreover, studies using animal models have suggested that the intestinal environment may play an essential role in aggressive behavior [19].

## 3. Intestinal Microbiota

### 3.1. Characteristics of the Intestinal Microbiota

The human gastrointestinal tract is inhabited by over 1 × 10^14^ microbes, including more than 1,000 bacterial types that are mostly located in the colon [20]. These bacterial species initiate metabolic activities that result in energy recovery in the form of short-chain fatty acids, vitamin K production, iron absorption, the regulation of epithelial cell proliferation and differentiation, immune system development, and protection against pathogens [21,22]. They are also believed to be deeply involved in host health, the regulation of fat accumulation, and stimulation of angiogenesis [23]. Although the composition of adult human gut microbiota is thought to be stable over time, it may vary markedly among individuals [24].

### 3.2. Developmental Processes of the Intestinal Microbiota

**Figure 1:**
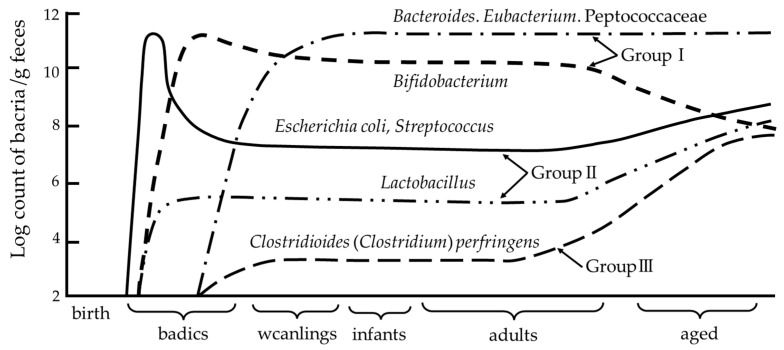
Age-related changes in intestinal microbiota.

The formation of gut microbiota begins immediately after birth [20,25]. The first bacteria to colonize the intestines of newborns are those of maternal origin delivered vaginally. The intestinal environment of newborns shows a positive redox potential at birth [20,26]. Thus, the gastrointestinal tract is first colonized by facultative anaerobes that diminish the redox potential and allow for the growth of facultative anaerobes, which usually appear in large numbers during the first week of life [20,26]. Thus, the first bacteria to establish and form gut microbiota are facultative anaerobes, such as Staphylococcus, Streptococcus, and Enterobacteriaceae (mainly *E. coli*). The presence of these bacteria is transient as they reduce the redox potential, enabling colonization by biased anaerobes and the emergence of Bacteroides and *Bifidobacterium* [26,27,28,29] (Figure 1). At approximately 1 month of age, *Bifidobacterium* is most predominant in the feces of bottle- and breast-fed infants [30]. Although *Bifidobacterium* ceases to be the predominant species in the intestine after weaning, it continues to be present in the intestine into old age. Bifidobacterium-dominated intestinal microflora in infants is considered as an indicator of a healthy intestinal tract, and the facultative anaerobes that initiate this dominance play an important role in priming *Bifidobacterium*. Thus, the neonatal gut microbiota that form continue to influence the host and its gut microbiota throughout life [22]. Clinical studies indicate that the early intestinal colony formation provides an enormous microbial stimulus that leads to profound changes in the development of intestinal and mucosal immune systems [31,32]. Therefore, microbial colonization during infancy plays a vital role in lifelong health maintenance.

This figure is from the study of Mitsuoka [27] with permission. The major bacteria detected in intestinal microbiota were roughly divided into three groups: (1) lactic acid bacteria, including *Bifidobacterium*, *Lactobacillus*, and *Streptococcus* (including *Enterococcus*); (2) anaerobic bacteria, including Bacteroidaceae, anaerobic curved rods, *Eubacterium*, Peptococcaceae, *Veillonella*, *Megasphaera*, *Gemmiger*, *Clostridioides* (*Clostridium*), and *Treponema*; and (3) aerobic bacteria, including Enterobacteriaceae, *Staphylococcus*, *Bacillus*, *Corynebacterium*, *Pseudomonas*, and *yeasts*. These bacterial groups are further divided into several species or biovars.

### 3.3. Role of Bifidobacteria

Bifidobacteria are beneficial intestinal bacteria that are present in the microbial colonies of infants. Bifidobacteria become the predominant microorganism in the intestine within the first week of life, and maintain their predominance until weaning (Figure 1). The functions performed by bifidobacteria include the production and release of antioxidants, the maturation of the immune system during childhood, the maintenance of both prenatal immune homeostasis and intestinal barrier function, and the protection against pathogens by inhibiting pathogen adhesion to intestinal mucosa [33]. Furthermore, studies using germ-free (GF) mice suggest that certain species and strains of bifidobacteria may reduce stress responses [34] and influence behavior and mental activity [35]. Another important function of the genus *Bifidobacterium,* which contributes to gut homeostasis and host health, is the production of acetic and lactic acids during carbohydrate fermentation, which are converted to butyric acid by other enteric bacteria via cross-feeding interactions [33,36]. Thus, the development of a healthy bifidobacterial flora in early childhood may play an important role in resistance to diseases later in life.

### 3.4. Effects of the Intestinal Environment on Behavior and Mental Activity

The gut and the brain transmit information bidirectionally via humoral factors, such as hormones and cytokines and the autonomic nervous system. Gut microbiota is recognized as an important component of this process, which is referred to as the “microbiota–gut–brain axis [37,38]”. Liver and gallbladder metabolism, immunomodulatory responses, neuronal innervation, intestinal secretion, and microbial metabolite signaling have been implicated in various known bidirectional communication pathways between the gut microbiota and brain [39].

Multiple mouse-based studies have indicated that gut bacteria and their metabolites can influence not only the stress response, but also behavioral and mental activities via the gut–brain axis [34,35,40,41]. When the body is exposed to toxic stress, the HPA axis and sympathetic nervous system are activated to maintain homeostasis. Sudo et al. [34] hypothesized that the development and maturation of the HPA axis, which constitutes a major biological defense response, is influenced not only by genetic factors, but also by the gut bacteria that become established shortly after birth. The authors compared the responses of GF and specific-pathogen-free (SPF) mice to restraint stress, and found that BALB/c GF mice showed significantly elevated responses to the adrenocorticotropic hormone and corticosterone, revealing that the composition of intestinal microbiota present immediately after birth may determine the differences in the responses shown to stress by the host when older. This finding triggered subsequent studies that investigated the effects of gut microbiota on mental activity and behavior. Nishino et al. [35] selected BALB/c GF mice and transplanted their pups (second generation; bred in isolators) into four groups: GF mice; mice orally administered with fecal dilutions from SPF mice (Ex-GF); GF mice with a single strain of *Bifidobacterium infantis* (*B. infantis*); and GF mice with a single strain of *Blautia coccoides* (*B. coccoides*). Each group was then bred, and these pups (third generation) were used for the analysis. Activities were evaluated by measuring the distance traveled using the open-field method, while anxiety-related behavior was evaluated by measuring the time spent in locomotion using the open-field method and glass ball hiding behavior. GF mice exhibited enhanced hyperactivity and anxiety symptoms compared to Ex-GF mice. In addition, when the GF group was compared with a single *B. infantis* or *B. coccoides* transplant, the *B. infantis*-transplanted group was less active than the GF mice group, although their anxiety levels were similar. However, the *B. coccoides*-transplanted group showed the same level of activity, but less anxiety than the GF group. These results suggested that the gut microbiota influences hyperactivity and anxiety symptoms, and that a specific species may be helpful in treating these.

## 4. Intestinal Environment and Aggression in Animals

Evidence showing the effects of gut microbiota on emotion led to the hypothesis that the gut microbiota could influence one of the oldest problem behaviors: aggression. Animal studies that assessed this hypothesis were summarized (Table 1).

### 4.1. Mice

Based on the findings of Nishino et al. [35], Watanabe et al. [45] tested the effects of gut microbiota on host aggression in a contamination-free environment. Here, male–female pairs of BALB/c GF mice were selected as the first generation, and their pups (second generation) were classified as either GF or Ex-GF. These second-generation mice were further bred and the males of each third-generation brood were used in the experiments as GF (n = 30) or Ex-GF (n = 30). At 8 weeks of age, Ex-GF and castrated Ex-GF mice were placed diagonally opposite each other in an open field box in a strictly uncontaminated isolator. The same experiment was repeated with GF and castrated GF mice. The GF mice exhibited more aggressive behavior than the Ex-GF mice. Additionally, the concentrations of dopamine (DA) and its metabolite, dihydroxyphenylacetic acid (DOPAC), in the prefrontal cortex, striatum, and brainstem of 10-week-old GF and Ex-GF mice (n = 9 for each) were measured immediately after aggression testing, via high-performance liquid chromatography. GF mice displayed higher concentrations of DA and lower concentrations of DOPAC in each region than Ex-GF mice, suggesting that GF mice had a reduced DA metabolic turnover capacity in these areas, indicating that a reduced brain DA transmission capacity was associated with aggressive behavior. Brain DA transmission is presumed to be an important regulator of the onset and development of aggressive behavior [61]. The results of Watanabe et al. [45] were compatible with those of Schlüter et al. [61].

Leclercq et al. [42] investigated the effect of administering an antibiotic (low-dose penicillin to mothers during late gestation and early postnatal (weaning) periods on the mental activity and behavior of mouse pups. BALB/c pups were treated with antibiotics (n = 25; 12 males and 13 females), antibiotic and *Lactobacillus rhamnosus* JB-1 (n = 19; 6 males and 13 females), or used as controls (n = 28; 11 males and 17 females). Exposure to antibiotics exerted a persistent effect on the gut microbiota of pups, increased expression of cytokines (IL-6 and Il-10) and chemokines (IL-8) in the frontal cortex, and modulated the integrity of the blood–brain barrier. Furthermore, mice exposed to antibiotics showed increased anxiety-like behavior and impaired social behavior. Pups whose mothers had been exposed to penicillin throughout gestation and weaning were more prone to aggression. These results suggested that the disruption of gut microbiota using antibiotics during childhood may affect behavior later in life.

### 4.2. Dogs

Kirchoff et al. [47] collected fecal samples from 31 pit-bull-type dogs (14 males and 17 females), including 21 that exhibited aggressive behavior and 10 that did not. Next-generation sequencing of the V3–V4 region of the bacterial 16S rRNA was performed on the intestinal microflora of samples. The beta diversity of intestinal microbiota differed between aggressive and normal dogs, supporting a link between canine aggression and the composition of intestinal microbiota. In addition, the relative abundances of several bacteria, including *Lactobacillus*, *Dorea*, *Blautia*, *Turicibacter*, and *Bacteroides,* in the gut microbiota of aggressive dogs were altered. This study indicated that gut microbiota may be useful for diagnosing aggression in dogs prior to the manifestation of potentially discerning cryptic etiologies of aggression.

Mondo et al. [48] used next-generation sequencing of the V3–V4 region of bacterial 16S rRNA in a study in which 42 dogs (23 males and 19 females) were classified into three behavioral groups: aggressive (n = 11), phobic (n = 13), and normal (n = 18); the gut microbiome of each group was compared. The results showed that, at the family level, Lachnospiraceae, Erysipelotrichaceae, and Clostridiaceae were the major components of the normal group (relative abundances >10%). In the aggressive group, Bacteriodaceae, Alcaligenaceae, and Paraprevotellaceae were significantly decreased and Erysipelotrichaceae was significantly increased compared with those in the normal group. At the genus level, Clostridioides (Clostridium), Lactobacillus, Blautia, and Collinsella were predominant in the normal group (relative abundance >5%) while the levels of *Oscillospira, Peptostreptococcus, Bacteroides, Sutterella,* and *Coprobacillus* spp., were significantly lower in the aggressive group compared with those of the normal group, whereas Catenibacterium, Megamonas, and Eubacterium showed an opposite trend. Behaviorally discriminatory bacterial genera in dogs with aggressive behavior were Catenibacterium and Megamonas. Furthermore, simultaneous measurements of testosterone and cortisol, hormones known to be involved in aggressive behavior, showed that the concentrations and ratios of these hormones did not differ between dogs showing aggressive and normal behavior. The results of this study suggested that the intestinal environment was more closely related to aggressive behavior than other behavioral abnormalities, and that metabolites derived from the intestinal microbiota and the microbiota itself may be more associated with aggression than hormonal effects.

### 4.3. Hamsters

Sylvia et al. [50] used male (n = 18) and female (n = 18) Siberian hamsters to evaluate the effects of single and repeated administrations of a broad-spectrum antibiotic (Abx) (Enrofloxacin) on aggression compared with a control group (n = 9 for both males and females). Hamsters were treated or not treated on days 1–7 of the experiment, and again on days 15-21 (with a 7 d recovery period); Abx animals received Abx, while the control animals received sterile water. On days 22–28 (second recovery period), all animals were monitored. Male hamsters treated with antibiotics for 7 d showed no change in overall aggression compared with male hamsters treated with sterile water for 7 d. On the other hand, female hamsters treated with antibiotics for 7 d showed a decrease in aggression compared with females treated with antibiotics for 7 d. Male hamsters that received two 7 d antibiotic treatments with a 7 d recovery period between them showed a decrease in overall aggression compared with males that received two control treatments. Under similar conditions, females were nearly identical. After two recovery periods, aggression returned to normal levels in males, but not in females. The results of this study suggested that antibiotic treatments with fluoroquinolones may have exerted a sex-dependent, sustained effect on the social behavior of hamsters and other rodents.

Ren et al. [54] examined the relationship between photoperiod-related aggression and gut microbiota in Siberian hamsters. Following a 1-week acclimation period, male and female hamsters were randomly assigned to either long-day (LD) (9 males and 9 females; light:dark, 16 h:8 h) or short-day (SD) (17 males and 17 females; light:dark, 8 h:16 h) groups. Among SD hamsters, those that lost more than 5% of their body weight and had gonadal regression were classified as short-day responders (SD-Rs), while those that did not meet these criteria were classified as short-day nonresponders (SD-NRs). Of the hamsters reared under SD conditions, 53% of both male and female hamsters responded physiologically to changes in the photoperiod (SD-R; nine males and nine females). Results showed that the duration of an attack, number of attacks, and time to first attack of SD-R males were not significantly different from those in SD-NR and LD males. By contrast, the attack duration and number of attacks in SD-R females were significantly different from those in SD-NR and LD females. At week 9, SD-R females had longer attack durations than LD and SD-NR females. SD-R females also exhibited a significantly higher number of attacks than SD-NR females at week 0 and all other groups in subsequent weeks. The relative abundance of Anaeroplasmataceae in SD-R (but not in LD and SD-NR) females at week 9 was positively correlated with attack frequency. This study indicated that the photoperiod was associated with sex-specific changes in gut microbiota and aggression, and that gut microbiota may be associated with the number and duration of attacks. Thus, the gut microbiota may constitute a component of the seasonal switch hypothesis.

Cusick et al. [55] allocated pregnant Siberian hamsters into four groups for the duration of gestation as follows: stress only (7 females and 9 males); antibiotics (enrofloxacin) only (10 females and 8 males); antibiotics and stress (7 females and 9 males); and control (8 female and 9 males). Then, their gut microbiome composition and diversity, stress-induced cortisol levels, and social behavior were quantitatively assessed. The aggression scores of offspring produced by stress only mothers differed significantly from offspring produced by antibiotic and stress mothers. The aggression scores of the offspring of control mothers also differed significantly from offspring produced by stress only mothers. The analysis of individual males and females showed that female offspring produced by stress only mothers were more aggressive than female offspring produced by both control and antibiotic and stress mothers. Female offspring of antibiotic and stress mothers were more similar to those of control mothers in their aggressive behavior, displaying low levels of aggression. By contrast, male offspring of stress only mothers displayed levels of aggression that were similar to that of the male offspring of control mothers. Unlike female offspring, the male offspring of antibiotic and stress and antibiotic only mothers were more aggressive than male offspring from other maternal treatment groups. This showed that maternal gut microbiota affected the aggressiveness of offspring, and that this effect differed between sexes.

Shor et al. [56] studied the relationship between the photoperiod, aggression, and gut microbiota in male Siberian hamsters using fecal microbiota transplants (FMTs) from donor hamsters. Hamsters were randomly assigned to four treatment groups: LDfs, hamsters housed in LD conditions receiving FMTs from an SD (fs) donor (n = 8); SDfl, hamsters housed in SD conditions receiving FMTs from an LD (fl) donor (n = 6); LDfl, hamsters housed in LD conditions receiving FMTs from an LD (fl) donor (n = 3); and SDfs, hamsters housed in SD conditions receiving FMTs from an SD (fs) donor (n = 3). The results indicated that latency to first attack was significantly longer in LD-housed hamsters that received LD microbiota (LDfl) compared with that of hamsters housed under SD conditions and having received SD microbiota (SDfs). The latency to attack in the groups that received opposing microbiota (LDfs and SDfl groups) was intermediate and did not differ from that of any other group. SD-housed hamsters receiving SD microbiota (SDfs) exhibited a greater duration of aggression than both LD groups. Implanting SD Siberian hamsters with fecal microbiota from LD hamsters resulted in a reversal of seasonal aggression, whereby SD hamsters displayed aggression levels typical of LD hamsters. The results implied that the gut microbiota may play a role in the photoperiodic mechanism regulating seasonal host behavior.

### 4.4. Drosophila

Jia et al. [58] investigated the relationship between *Drosophila* aggression and gut microbiota in conventionally reared (CR), GF flies, and GF embryos with mixed bacteria (MB) upon sterilization. The authors compared the frequency of lunging and the latency of fighting to initiate lunging in males, as well as the frequency of head butting and the latency to initiate head butting in females. GF–GF male pairs displayed a significantly reduced lunging frequency and delayed time to initiate lunging compared with that displayed by CR–CR pairs. The GF–CR male pairs exhibited decreased lunging frequency and an increase in fighting latency compared with those of CR–CR pairs. This may be attributed to reduced aggression in GF males. When MB males were assessed, they exhibited levels of aggression that were much higher than those of GF males, but were comparable to those of CR males. On the other hand, GF–GF female pairs showed a significantly lower head-butting frequency and significantly longer latency for head-butting initiation compared with those of CR–CR or MB–MB pairs. These results indicated that microbiota promoted aggressive behavior in both males and females. There was no difference between the courtship behavior of GF and MB males, but the mating success of MB males was higher. GF males exhibited a substantial decrease in intermale aggression, which was rescued using microbial recolonization. GF males were not as competitive as wildtype males in terms of mating, although they displayed regular levels of courtship behavior.

Grinberg et al. [59] investigated the association between aggression and the gut environment in male *Drosophila* by comparing flies grown on media supplemented with a mixture of antibiotics (Abx), *Lactobacillus brevis* (*L. brevis*)-monocolonized flies, *Lactobacillus plantarum (L. plantarum)*-monocolonized flies, and untreated flies (n = 8 for each). The Abx treatment significantly increased the number of aggressive encounters among male flies by nearly 150% compared with that of the control group, whereas supplementation with a single bacterial species (*L. plantarum* or *L. brevis*) significantly reduced aggression, compared with that seen in Abx-treated flies. This finding directly contradicted that of Jia et al. [58], who reported that aggression in GF flies was reduced. However, the effects of a sterile gut environment in the host may not be identical to that of an antibiotic-disturbed gut environment, so their results may have been inconsistent. Thus, a further validation of the results of these two studies may be warranted.

## 5. Therapeutic Intervention for Aggression Targeting Gut Microbiota

### 5.1. Normalized Gut Microbiota in GF Mice

Certain forms of human aggression are considered pathological behaviors that place a significant burden on society [3]. If left untreated, aggression can have serious consequences for individuals. Therefore, there is an urgent need for early and appropriate intervention. Although a few studies that targeted host gut microbiota to alter aggression do currently exist, recent years have seen a marked increase in interest towards treating aggression by targeting gut microbiota [19,62,63].

Watanabe et al. evaluated the highly aggressive behavior shown by GF mice in the above-mentioned study [45] and then therapeutically targeted their aggressive behavior by normalizing their intestinal microbiota [45]. Diluted feces from the same 8-week-old Ex-GF mice were orally administered to GF mice offspring at 0 (CVL 0, n = 10), 6 (CVL 6, n = 10), and 10 weeks (CVL 10, n = 9) of age, with aggressive behavior compared at 12 weeks. Mice inoculated at 0 and 6 weeks showed less aggression than mice inoculated at 10 weeks of age. Furthermore, aggression exhibited by mice in the CVL 6 and CVL10 groups did not differ from that exhibited by 8-week-old GF mice, but mice in the CVL 0 group were less aggressive than GF mice. These findings indicated that the normalization of gut microbiota reduced the aggression in mice, and that the effect was greater earlier in development, suggesting that the maintenance of a healthy gut microbiota during early developmental stages may contribute to reduced host aggression.

### 5.2. Probiotics and Prebiotics

If host aggression is attributable to disruptions in intestinal microbiota, then probiotics and prebiotics should be successful as therapeutic interventions. Probiotics are live microorganisms that, when administered in adequate amounts, confer health benefits on the host [64]; most probiotics are bacterial species (e.g., *Bifidobacterium* and *Lactobacillus* spp.) [64]. Probiotics help maintain a healthy digestive tract by promoting a beneficial intestinal bacterial community, strengthening and regenerating intestinal mucosal cells, and producing short-chain fatty acids (SCFAs), such as acetic acid, propionic acid, and butyric acid. In addition, they supported a healthy immune system by preventing allergies, reducing inflammation, and enhancing anti-infection activity [64].

Prebiotics are substrates that can be selectively utilized by host microorganisms, conferring a health benefit [65]. Probiotics exert their effects by secreting SCFAs, which modulate certain metabolic activities, including colonocyte function, gut homeostasis, energy gain, the immune system, blood lipids, appetite, and renal physiology [65,66,67]. An important implication of the selective use of prebiotics by host microbiota is that it augments the production and secretion of SCFAs in the colon (humans) and cecum (rodents); >95% of these SCFAs are acetic, propionic, and butyric acids [65]. Specifically, butyrate is useful both inside and outside the intestine [68], and the major butyrate-producing bacteria include *Faecalibacterium prausnitzii*, *Eubacterium rectale*, and *Roseburia* [33].

Dietary prebiotics that are known to exert health benefits on humans are nondigestible oligosaccharides, fructooligosaccharides (FOS), galactooligosaccharides (GOS), and inulin, which is classified as a dietary fiber [65]. Bifidobacteria, the dominant bacterial species in the gut, contain β-fructanosidase and β-galactosidase, which help selectively metabolize FOS and GOS, not only increasing the ratio of bifidobacteria in the gut, but also contributing to the production of lactic acid and acetic acid from bifidobacteria. Cross-feeding involves the partial hydrolysis of a carbohydrate substrate by primary degrading bacteria followed by the use of these carbohydrate degradation products as secondary substrates by other bacteria [36]. Butyrate-producing bacteria produce butyrate using either acetic acid, or both prebiotics and acetic acid, as substrates [33]. Therefore, acetic acid produced by bifidobacteria plays an important role as an intermediate in the cross-feeding of butyrate-producing bacteria [69,70]. Prebiotics such as FOS and GOS suggest a beneficial role for stress-related behavior. C57BL/6J male mice were given FOS, GOS, or a combination of FOS+GOS for 3 weeks prior to testing [71]. Exposure to the chronic prebiotic FOS+GOS showed effects that were both antidepressant and anxiolytic. In addition, GOS and the FOS+GOS combination reduced the stress-related corticosterone release. Concerning short-chain fatty acid concentrations, the prebiotic administration increased cecal acetate and propionate and reduced isobutyrate concentrations, changes that significantly correlated with the positive effects observed on behavior [71].

Probiotics and prebiotics play an important role in gut microbiota improvement. However, clear demonstrations of the therapeutic effects of probiotics and prebiotics on aggression in animals remain scant. To our knowledge, no studies have investigated the effects of intervening in the gut microbiota composition with aggression as the primary outcome, although a few have examined the effects on aggression as a secondary outcome. The neurodevelopmental disorder autism spectrum disorder (ASD) is characterized by deficits in social communication and interactions, as well as restricted and stereotypic behavior [72]. In addition, children and adolescents with ASD often display behavioral issues, such as irritability and aggression, which may manifest as tantrums, self-injury, and aggressive behavior toward others [73]. The approximate percentage of behavioral problems seen in ASD individuals, categorized as moderate or severe by parents and teachers, were 20% for irritability and exposure, 30% for temper tantrums, and 50–60% for easily frustrated emotionally [74]. Individuals with ASD often experience gastrointestinal disturbances, such as diarrhea and constipation [75], and intervention studies using probiotics and prebiotics in ASD patients [76] may help determine the effectiveness of gut microbiota intervention in reducing aggression. An aberrant behavior checklist (ABC) is most frequently used to assess aggression in neurodevelopmental disorders [77]. ABC is a parent-rated instrument, consisting of five subscales measuring irritability, agitation, and crying (15 items); lethargy/social withdrawal (16 items); stereotypic behavior (7 items); hyperactivity/noncompliance (16 items); and inappropriate speech (4 items), where higher scores indicate a greater severity [77].

Although a randomized trial reported that probiotic formulations reduced the severity of ASD symptoms, there were no significant differences between the behavioral problems or symptom severities seen in the probiotic and placebo groups [78,79,80,81,82]. Sanctuary et al. [83] compared the effects of prebiotics alone and prebiotics plus probiotics in a double-blind, crossover, randomized trial on 2- to 11-year-old ASD children (n = 8) with comorbid digestive symptoms. Oligosaccharides were used as the prebiotics and *B. infantis* as the probiotic. The study ran for 12 weeks, with both probiotics and prebiotics administered during the first 5 weeks, followed by a 2-week washout period, and then only prebiotics for the second 5 weeks. Before/after comparisons indicated that ABC-based irritability, stereotypy, hyperactivity, and the total score were significantly reduced in the prebiotic monotherapy group. By contrast, a pre/postcomparison in the combined prebiotic and probiotic group showed that only ABC lethargy was significantly decreased. Although most scores between the two groups were not significantly different, the prebiotic monotherapy group showed a significant improvement in regular behavior compared with the prebiotic and probiotic combination group. ABC scores decreased significantly in the prebiotic monotherapy group. However, this was only a before/after comparison and not a comparison with the control group.

### 5.3. Fecal Microbiota Transplant

Therapeutic intervention in gut microbiota using FMTS has recently received international attention. Here, a solution containing fecal matter from a healthy donor was administered to the recipient’s intestinal tract to elicit a substantial and sustainable restoration of the recipient’s microbiota. FMT samples were effective in treating recurrent Clostridioiedes difficile infections and, thus, were considered a promising treatment for chronic inflammatory diseases, including inflammatory bowel disease [84].

Kang et al. [85] conducted an open-label clinical trial involving 18 ASD patients (7–16 years) with moderate to severe gastrointestinal disorders. The FMT protocol for this study included 14 d of oral vancomycin and a random oral or transrectal administration of high-dose standardized human gut microbiota. After 10 weeks of treatment, the patients were followed up for another 8 weeks. The childhood autism rating scale (CARS), which assesses ASD core symptom severity, decreased significantly (by 22%) from the start to the end of treatment and by 24% after 8 weeks without treatment, compared to the baseline. ASD children showed improvements in total ABC scores, compared with those of the baseline, both at the end of the treatment (10 weeks) and after 18 weeks. These same 18 patients were followed up with for 2 years after completion [86]. The CARS results showed that ASD severity at the 2-year follow-up was 47% lower than the baseline, compared with the 22% reduction at the end of the 10-week treatment. Regarding the ABC, the total score continued to improve and was 35% lower than the baseline in the open-label intervention trial (it was 24% lower than the baseline at the end of the initial study). The percentage change in CARS and ABC scores was positively correlated with the percentage change in the gastrointestinal symptom rating scale scores, suggesting that the relief of gastrointestinal symptoms provided by the FMTs may improve behavioral severity in ASD children.

The FMTs showed potential for relieving ASD symptoms. However, in this study, the behavioral evaluation was small, while a placebo control, randomization, and blinding were absent. Thus, future validation via a large, double-blinded, placebo-controlled randomized trial is required. In addition, the donor FMT mixture was composed of many unknown taxa, including bacteria, yeasts, parasites, and viruses, which may exert beneficial effects, but also posed unknown risks via elevated antibiotic resistance and the production of genotoxic metabolites via intestinal transfer. The International Scientific Association for Probiotics and Prebiotics removed FMTs from its probiotic framework due to uncertainties regarding the amounts, types, and efficacies of the bacterial components present [64]. To develop FMTs into a standard treatment for ASD, multiple double-blind, placebo-controlled, randomized trials using fecal samples validated for efficacy and safety must be performed.

### 5.4. Potential Applications for Therapeutic Interventions Early in Development

A considerable number of animal-based studies have indicated that gut microbiota may influence aggression [42,45,47,48,50,54,55,56,58,59] and that therapeutic gut microbiota interventions targeting aggression are more likely to be effective when administered as early in development as possible [45]. Since most FMT trials are conducted using adults, data and knowledge regarding the effects of FMT on younger children remain limited, and, thus, the FDA restricted the Kang et al. study to older children aged 7 to 17 years [85]. FMTs are not intended to be administered to younger children due to their strict safety profile. As a result, most therapeutic interventions in the intestinal microbiota of younger children may have to be performed using probiotics and/or prebiotics. SCFAs are effective, active components secreted by probiotics [64]. The selective use of prebiotics by host microorganisms leads to the production and secretion of SCFAs [65,66,67]. Clinical studies using one of these two as a therapeutic tool were not fully successful. This may be attributed to issues related to the content, timing, and duration of administration. We propose that future applications be limited to infants in the early stages of development, and that both probiotics (mainly bifidobacteria) and prebiotics be simultaneously administered for a sufficiently long time period (more than 3 months).

### 5.5. Maternal Gut Microbiota

Due to therapeutic interventions in the gut microbiota during early development being effective in reducing aggression, it is possible that intervening in gut microbiota during the gestational stage would effectively reduce aggression in offspring, which may amount to the ultimate early intervention [19]. The dysbiosis of the maternal gut microbiota is increasingly being linked to abnormalities in brain function and behavior of offspring [87].

Leclercq et al. [42] reported that BALB/c mouse pups of mothers exposed to penicillin during the gestation to weaning period tended to exhibit aggressive behavior. Sylvia et al. [50] and Cusick et al. [55] reported that maternal exposure to antibiotics had induced aggressive behavior in Siberian hamsters. Watanabe et al. [45] bred male–female pairs of BALB/c GF mice (first generation) and assigned the second-generation mice to GF and Ex-GF groups. The third-generation mice bred from both groups were compared as GF and Ex-GF groups; the Ex-GF group showed less aggression. These results showed that Ex-GF mouse pups (third generation) of mothers (the second-generation Ex-GF mice) with normalized intestinal microflora would be less aggressive, indicating that the intestinal environment of the mother may exacerbate, attenuate, or even prevent aggression in pups.

Thus, if the mother’s gut environment influences aggression in her offspring, then an investigation into which factors in the mother’s gut microbiota determine the gut microbiota of the offspring is warranted [19]. Intestinal microbiota are formed immediately after birth, with the prototype of an individual’s intestinal microbiota being formed during the first week. The intestinal microbiota formed at this stage exert a significant impact on the lifelong intestinal microbiota [22]. Therefore, the intestinal environment of the mother is extremely important for determining the intestinal microbiota of her offspring [19]. Due to Bifidobacteria being the most abundant and important bacterial species in infants [33], healthy innate bifidobacteria are key to the formation of stable gut microbiota in newborns.

Mikami et al. [88] investigated the conditions under which maternal intestinal and vaginal microbiota would facilitate the formation of stable gut microbiota in newborns, with particular reference to *Bifidobacterium*. Mother–infant stool pair samples (n = 110) and birth canal secretions (n = 100) were analyzed using qualitative and real-time PCR methods. The results revealed that the presence of *Bifidobacterium breve* in maternal gut microbiota positively affected the abundance and diversity of *Bifidobacterium* in infant gut microbiota, while that of *B. infantis* only affected the abundance. This study showed that the presence of *B. breve* or *B. infantis* in the gut microbiota of pregnant women was associated with a healthier infant gut environment in their offspring. Sirilun et al. [89] assayed fecal samples from 120 healthy pregnant mothers and their 1-month-old infants one month after delivery, as well as 98 vaginal swabs from the mothers at the time of delivery using real-time PCR to detect *Bifidobacterium* species and estimate bifidobacterial copy numbers. When adjusted for the number of each *Bifidobacterium* species, the mode of delivery, and antibiotic use by infants up to 1 month of age, there was a significant association between the total number of *Bifidobacterium* species in the feces of mothers and the increase in the copy number of *Bifidobacterium* species in the feces of breast-fed infants. There was no significant correlation between the number of bifidobacteria copies in vaginal swabs and the number of bifidobacteria copies in the feces of the infants. These results suggested a significant association between the number of bifidobacteria in the guts of mothers and infants.

To verify the vertical transmission of bifidobacteria from mothers to newborns, by proving that a mother transmits her own unique bifidobacteria to her infant shortly after birth, the bifidobacteria of both mothers and their babies must be matched at a strain level. Takahashi et al. [90] hypothesized that *B. breve* was more likely to be vertically transmitted from mother to infant and verified this hypothesis at the strain level. Cultured strains were collected from each pair of stool samples in which a matched *B. breve* species was detected between the mother and offspring and both *B. breve* strains were investigated via the random amplification of polymorphic DNA techniques to examine whether they were matched between mother and offspring. The control group consisted of paired stool samples in which the mother’s most dominant species, *B. longum*, was detected consistently between mother and offspring. A comparison of the vertical transmission statuses of *B. breve* and *B. longum* strains suggested that *B. breve* strains were more likely to be vertically transmitted. Makino et al. used multilocus sequence typing (MLST) to investigate the vertical transmission of intestinal bifidobacterial strains between mothers and infants [91,92,93]. *Bifidobacterium* strains were isolated from fecal samples of 17 healthy mother–child pairs (12 vaginal deliveries and 5 caesarean sections) [91]. Fecal samples were collected twice from mothers before delivery and from infants at 0, 3, 7, 30, and 90 days of age. Bifidobacteria were isolated from these samples and classified using MLST; 273 *Bifidobacterium* strains and 5 *Bifidobacterium* species (*Bifidobacterium adolescentis*, *Bifidobacterium bifidum*, *Bifidobacterium catenulatum*, *B. longum subspecies longum*, and *Bifidobacterium pseudocatenulatum*) were isolated from both mothers and their offspring, revealing that they were monophyletic. These studies on the vertical transmission of bifidobacterial strains from mother-to-child indicated that bifidobacterial strains are transmitted from pregnant women to their infants. Thus, the bifidobacterial gut environment of pregnant women may play an important role in the development of healthy gut microbiota in infants, and stable gut microbiota in the mother may influence the healthy gut microbiota of offspring and, consequently, reduce aggression.

## 6. Conclusions

Although interventions in the gut microbiota of patients with ASD using probiotics, prebiotics, or FMTs showed therapeutic potential for reducing aggression as a secondary outcome, clear evidence-based clinical studies that target gut microbiota with aggression as the primary outcome are currently unavailable. Recent studies using mouse, dog, hamster, and drosophila models have indicated that the intestinal environment affects aggression. In addition, our GF mice study indicated that a gut microbiota intervention at the earliest possible developmental stage yields the most effective reduction in aggression. The use of animal models and clinical trials to examine the effects on aggression as a secondary outcome has yielded evidence indicating that gut microbiotas do influence aggression. The FMT treatment should be reserved for younger children due to the safety requirements. The most promising therapeutic intervention in the gut microbiota of younger children during early developmental stages appeared to be intervention using probiotics and/or prebiotics. We propose that the future direction of treating aggression should be aimed only at younger children in early developmental stages and that both probiotics, mainly bifidobacteria, and prebiotics should be used simultaneously and administered for a sufficiently long period of time. Studies investigating therapeutic interventions for aggression based on the gut microbiota are still in the formative stages. Both animal-based and clinic-based research aimed at clarifying the causal relationship between gut microbiota and aggression may be needed prior to the proper clinical application of therapeutic intervention in gut microbiotas.

## Figures and Tables

**Table 1:** Research on aggressive behaviors associated with gut microbiota in animals.

Reference	Animal Model	Main Comparable or Exposure	Assessment of Aggression	Effect of Gut Microbiota on Aggression
Leclercq et al. [42]	Mouse	Pregnant females were treated with either drinking water (control group), antibiotics, or antibiotics and Lactobacillus until weaning of pups (postnatal day 21).	Short (acute) version adapted from the chronic social defeat described by Krishnan et al. [43] and Golden et al. [44] was assessed.	Mice pups, whose gut microbiotas were disturbed with antibiotics from gestation through weaning, showed increased anxiety-like and impaired social behaviors, as well as a tendency to exhibit aggression.
Watanabe et al. [45]	Mouse	Germ-free (GF) and Ex-germ-free (EX-GF) mice	Aggressive behaviors including biting, wrestling, tail-rattling, aggressive grooming, or chasing were assessed based on the study by Schneider et al. [46]	Ex-GF mice with commensal gut microbiota showed significantly lower levels of aggression-related behavior than GF mice. When GF mice were conventionalized by administering feces from Ex-GF mice, the groups administered feces at 0 and 6 weeks of age showed aggression behaviors less frequently than normal GF mice. Among these mice conventionalized with Ex-GF feces, both the aggressive behavior rates of mice at 0 and 6 weeks were significantly lower than at 10 weeks.
Kirchoff et al. [47]	Dog	Aggressive and nonaggressive dogs	Aggressive dogs displayed aggression during one of three scenarios: an introduction to a life-size dog plush, introduction to a dog of the same-sex behind a barrier, and introduction to a dog of the same-sex without a barrier. Aggressive displays toward the life-size dog plush included growling, snarling, biting, holding, and shaking combined with tense behavior inconsistent with object play, as well as aggressive displays toward the same-sex dogs including growling and lunging, lunging and snarling, climbing on withers and growling, attempting to bite, and biting.	Differences in the β diversity of the gut microbiota between aggressive and nonaggressive dogs supported a link between canine aggression and the composition of the gut microbiota. In addition, several bacteria (Lactobacillus, Dorea, Blautia, Turicibacter, and Bacteroides) had increased and decreased relative abundances in aggressive dogs compared to nonaggressive dogs.
Mondo et al. [48]	Dog	Dogs with aggressive, phobic, or normal behavior	Aggressive behaviors were evaluated based on the study by Giussani et al. [49].	The relative abundance of commonly classified subdominant bacteria, such as *Catenibacterium* and *Megamonas,* was increased in the gut microbiota of dogs exhibiting aggressive behavior. Levels of testosterone and cortisol, hormones involved in aggressive behavior, were not closely associated with gut microbiota.
Sylvia et al. [50]	Hamster	Hamsters that received antibiotics or sterilized water	Using the resident–intruder paradigm with same-sex social partners per previously outlined methods for this species, aggressive behaviors were assessed based on the studies by Jasnow et al. [51], Rendon et al. [52], and Rendon et al. [53].	The effects of single versus repeated antibiotic treatments (including a recovery phase) on behavior were tested. Two treatments caused marked decreases in aggressive behavior in males; aggression returned to normal levels following recovery. Antibiotic-treated females, in contrast, showed decreased aggression after a single treatment. Unlike males, female aggression did not return to normal during either recovery period.
Ren et al. [54]	Hamster	Hamsters housed in either long-day or short-day (SD) photoperiods for 9 weeks. SD conditions were divided into short-day responders (SD-R) and short-day nonresponders (SD-NR) according to physiological response to changes in the photoperiod.	Aggressive behaviors including latency to first attack, frequency and duration of attacks, and chases were assessed based on the studies by Jasnow et al. [51], Rendon et al. [52], Rendon et al. [53], and Sylvia et al. [50].	SD-R females displayed increased aggression. The relative abundance of anaeroplasmataceae in females was associated with aggression in SD-R hamsters.
Cusick et al. [55]	Hamster	Pregnant females were assigned to one of four treatments: antibiotics only, stress only, antibiotics and stress, or control.	The resident–intruder trial, the frequency and duration of actions performed by the experimental individuals (i.e., residents), was examined and scored, following an established protocol in the authors’ lab. Aggressive (e.g., attack, chase) and nonaggressive (e.g., intruder investigation) behaviors were assessed.	Considering males and females individually, female offspring produced by stress only mothers were more aggressive than both female offspring produced by control mothers and female offspring produced by antibiotic and stress mothers. Maternal exposure to antibiotics affected the aggressive behaviors of male offspring.
Shor et al. [56]	Hamster	Hamsters were randomly assigned into four treatment groups: LDfs, hamsters housed in long-day (LD) conditions, which received fecal microbiota transplants (FMTs) from a short-day (SD) (fs) donor; SDfl, hamsters housed in SD conditions that received FMT from an LD (fl) donor; LDfl, hamsters housed in LD conditions that received FMT from an LD (fl) donor; SDfs, hamsters housed in SD conditions that received FMT from an SD (fs) donor.	The resident–intruder (R–I) paradigm was conducted to quantify the effects of FMT on aggressive behavior. The R–I procedure involved placing an “intruder” animal into the home cage of a “resident” animal and observing the subsequent displays of aggressive behaviors [57].	Implanting short-day Siberian hamsters with fecal microbiota from LD hamsters resulted in a reversal of seasonal aggression, whereby SD hamsters displayed aggression levels typical of LD hamsters.
Jia et al. [58]	Drosophila	Conventionally reared and germ-free (GF) flies, and GF embryos with mixed bacteria	Number of lunges in males and head butting in females was manually counted. For intermale aggression, lunging frequency and latency of fighting (the time from the beginning to the first lunging) were used to compare aggression levels.	The microbiota promoted aggressive behaviors in both Drosophila males and females. GF males showed a substantial decrease in inter-male aggression, which was rescued using microbial recolonization. These germ-free males were not as competitive as wildtype males for mating with females, although they displayed regular levels of courtship behaviors.
Grinberg et al. [59]	Drosophila	Flies grown on media supplemented with a mixture of antibiotics (Abx) to eliminate gut bacteria, Lactobacillus brevis-monocolonized flies, Lactobacillus plantarum-monocolonized flies, or untreated flies (control). Each group consisted of eight males.	Under ideal conditions for aggression [60], the visible movements of lunging, boxing, chasing, or wing threats were counted.	The Abx treatment increased the number of aggressive encounters among male flies compared to the control group, whereas supplementation with L. plantarum or L. brevis reduced aggression compared to both the Abx-treated flies.

This table was modified from the table published in an article by Mikami et al. [19].

## Data Availability

Not applicable.

## References

[1] Anderson D.J. (2012). Optogenetics, sex, and violence in the brain: Implications for psychiatry. Biol. Psychiatry.

[2] De Boer S.F. (2018). Animal models of excessive aggression: Implications for human aggression and violence. Curr. Opin. Psychol..

[3] Flanigan M.E., Russo S.J. (2019). Recent advances in the study of aggression. Neuropsychopharmacology.

[4] Siever L.J. (2008). Neurobiology of aggression and violence. Am. J. Psychiatry.

[5] Hsu Y., Earley R.L., Wolf L.L. (2006). Modulation of aggressive behaviour by fighting experience: Mechanisms and contest outcomes. Biol. Rev. Camb. Philos. Soc..

[6] Aleyasin H., Flanigan M.E., Russo S.J. (2018). Neurocircuitry of aggression and aggression seeking behavior: Nose poking into brain circuitry controlling aggression. Curr. Opin. Neurobiol..

[7] Mahadevia D., Saha R., Manganaro A., Chuhma N., Ziolkowski-Blake A., Morgan A.A., Dumitriu D., Rayport S., Ansorge M.S. (2021). Dopamine promotes aggression in mice via ventral tegmental area to lateral septum projections. Nat. Commun..

[8] Caramaschi D., de Boer S.F., de Vries H., Koolhaas J.M. (2008). Development of violence in mice through repeated victory along with changes in prefrontal cortex neurochemistry. Behav. Brain Res..

[9] De Boer S.F., Caramaschi D., Natarajan D., Koolhaas J.M. (2009). The vicious cycle towards violence: Focus on the negative feedback mechanisms of brain serotonin neurotransmission. Front. Behav. Neurosci..

[10] Calcagnoli F., de Boer S.F., Beiderbeck D.I., Althaus M., Koolhaas J.M., Neumann I.D. (2014). Local oxytocin expression and oxytocin receptor binding in the male rat brain is associated with aggressiveness. Behav. Brain Res..

[11] Koolhaas J.M., de Boer S.F., Coppens C.M., Buwalda B. (2010). Neuroendocrinology of coping styles: Towards understanding the biology of individual variation. Front. Neuroendocrinol..

[12] Haller J., Mikics E., Halász J., Tóth M. (2005). Mechanisms differentiating normal from abnormal aggression: Glucocorticoids and serotonin. Eur. J. Pharmacol..

[13] Heinz A.J., Beck A., Meyer-Lindenberg A., Sterzer P., Heinz A. (2011). Cognitive and neurobiological mechanisms of alcohol-related aggression. Nat. Rev. Neurosci..

[14] Miczek K.A., Weerts E.M., Tornatzky W., DeBold J.F., Vatne T.M. (1992). Alcohol and “bursts” of aggressive behavior: Ethological analysis of individual differences in rats. Psychopharmacology.

[15] Chiavegatto S., Quadros I.M., Ambar G., Miczek K.A. (2010). Individual vulnerability to escalated aggressive behavior by a low dose of alcohol: Decreased serotonin receptor mRNA in the prefrontal cortex of male mice. Genes Brain Behav..

[16] Veenema A.H. (2009). Early life stress, the development of aggression and neuroendocrine and neurobiological correlates: What can we learn from animal models?. Front. Neuroendocrinol..

[17] Haller J., Harold G., Sandi C., Neumann I.D. (2014). Effects of adverse early-life events on aggression and anti-social behaviours in animals and humans. J. Neuroendocrinol..

[18] Tóth M., Halász J., Mikics E., Barsy B., Haller J. (2008). Early social deprivation induces disturbed social communication and violent aggression in adulthood. Behav. Neurosci..

[19] Mikami K., Tochio T., Watanabe N., Martin C., Preedy V.R., Patel V.B. (2023). Modeling aggression in animals. Handbook of Anger, Aggression, and Violence.

[20] Wallace T.C., Guarner F., Madsen K., Cabana M.D., Gibson G., Hentges E., Sanders M.E. (2011). Human gut microbiota and its relationship to health and disease. Nutr. Rev..

[21] Guarner F., Malagelada J.R. (2003). Gut flora in health and disease. Lancet.

[22] Mikami K., Kimura M., Takahashi H. (2012). Influence of maternal bifidobacteria on the development of gut bifidobacteria in infants. Pharmaceuticals.

[23] Eckburg P.B., Bik E.M., Bernstein C.N., Purdom E., Dethlefsen L., Sargent M., Gill S.R., Nelson K.E., Relman D.A. (2005). Diversity of the human intestinal microbial flora. Science.

[24] Faith J.J., Guruge J.L., Charbonneau M., Subramanian S., Seedorf H., Goodman A.L., Clemente J.C., Knight R., Heath A.C., Leibel R.L. (2013). The long-term stability of the human gut microbiota. Science.

[25] Favier C.F., de Vos W.M., Akkermans A.D. (2003). Development of bacterial and bifidobacterial communities in feces of newborn babies. Anaerobe.

[26] Bezirtzoglou E. (1997). The intestinal microflora during the first weeks of life. Anaerobe.

[27] Mitsuoka T. (1992). Intestinal flora and aging. Nutr. Rev..

[28] Mackie R.I., Sghir A., Gaskins H.R. (1999). Developmental microbial ecology of the neonatal gastrointestinal tract. Am. J. Clin. Nutr..

[29] Caicedo R.A., Schanler R.J., Li N., Neu J. (2005). The developing intestinal ecosystem: Implications for the neonate. Pediatr. Res..

[30] Benno Y., Sawada K., Mitsuoka T. (1984). The intestinal microflora of infants: Composition of fecal flora in breast-fed and bottle-fed infants. Microbiol. Immunol..

[31] Lundell A.C., Björnsson V., Ljung A., Ceder M., Johansen S., Lindhagen G., Törnhage C.J., Adlerberth I., Wold A.E., Rudin A. (2012). Infant B cell memory differentiation and early gut bacterial colonization. J. Immunol..

[32] Olszak T., An D., Zeissig S., Vera M.P., Richter J., Franke A., Glickman J.N., Siebert R., Baron R.M., Kasper D.L. (2012). Microbial exposure during early life has persistent effects on natural killer T cell function. Science.

[33] Rivière A., Selak M., Lantin D., Leroy F., De Vuyst L. (2016). Bifidobacteria and butyrate-producing colon bacteria: Importance and strategies for their stimulation in the human gut. Front. Microbiol..

[34] Sudo N., Chida Y., Aiba Y., Sonoda J., Oyama N., Yu X.N., Kubo C., Koga Y. (2004). Postnatal microbial colonization programs the hypothalamic-pituitary-adrenal system for stress response in mice. J. Physiol..

[35] Nishino R., Mikami K., Takahashi H., Tomonaga S., Furuse M., Hiramoto T., Aiba Y., Koga Y., Sudo N. (2013). Commensal microbiota modulate murine behaviors in a strictly contamination-free environment confirmed by culture-based methods. Neurogastroenterol. Motil..

[36] De Vuyst L., Leroy F. (2011). Cross-feeding between bifidobacteria and butyrate-producing colon bacteria explains bifdobacterial competitiveness, butyrate production, and gas production. Int. J. Food Microbiol..

[37] Collins S.M., Bercik P. (2009). The relationship between intestinal microbiota and the central nervous system in normal gastrointestinal function and disease. Gastroenterology.

[38] Bercik P., Denou E., Collins J., Jackson W., Lu J., Jury J., Deng Y., Blennerhassett P., Macri J., McCoy K.D. (2011). The intestinal microbiota affect central levels of brain-derived neurotropic factor and behavior in mice. Gastroenterology.

[39] Cryan J.F., O’Riordan K.J., Cowan C.S.M., Sandhu K.V., Bastiaanssen T.F.S., Boehme M., Codagnone M.G., Cussotto S., Fulling C., Golubeva A.V. (2019). The microbiota-gut-brain axis. Physiol. Rev..

[40] Neufeld K.M., Kang N., Bienenstock J., Foster J.A. (2011). Reduced anxiety-like behavior and central neurochemical change in germ-free mice. Neurogastroenterol. Motil..

[41] Vuong H.E., Yano J.M., Fung T.C., Hsiao E.Y. (2017). The microbiome and host behavior. Annu. Rev. Neurosci..

[42] Leclercq S., Mian F.M., Stanisz A.M., Bindels L.B., Cambier E., Ben-Amram H., Koren O., Forsythe P., Bienenstock J. (2017). Low-dose penicillin in early life induces long-term changes in murine gut microbiota, brain cytokines and behavior. Nat. Commun..

[43] Krishnan V., Han M.H., Graham D.L., Berton O., Renthal W., Russo S.J., Laplant Q., Graham A., Lutter M., Lagace D.C. (2007). Molecular adaptations underlying susceptibility and resistance to social defeat in brain reward regions. Cell.

[44] Golden S.A., Christoffel D.J., Heshmati M., Hodes G.E., Magida J., Davis K., Cahill M.E., Dias C., Ribeiro E., Ables J.L. (2013). Epigenetic regulation of RAC1 induces synaptic remodeling in stress disorders and depression. Nat. Med..

[45] Watanabe N., Mikami K., Hata T., Kimoto K., Nishino R., Akama F., Yamamoto K., Sudo N., Koga Y., Matsumoto H. (2021). Effect of gut microbiota early in life on aggressive behavior in mice. Neurosci. Res..

[46] Schneider R., Hoffmann H.J., Schicknick H., Moutier R. (1992). Genetic analysis of isolation-induced aggression. I. Comparison between closely related inbred mouse strains. Behav. Neural Biol..

[47] Kirchoff N.S., Udell M.A.R., Sharpton T.J. (2019). The gut microbiome correlates with conspecific aggression in a small population of rescued dogs (*Canis familiaris*). PeerJ.

[48] Mondo E., Barone M., Soverini M., D’Amico F., Cocchi M., Petrulli C., Mattioli M., Marliani G., Candela M., Accorsi P.A. (2020). Gut microbiome structure and adrenocortical activity in dogs with aggressive and phobic behavioral disorders. Heliyon.

[49] Gaiani R., Chiesa F., Mattioli M., Nannetti G., Galeati G. (1984). Androstenedione and testosterone concentrations in plasma and milk of the cow throughout pregnancy. J. Reprod. Fertil..

[50] Sylvia K.E., Jewell C.P., Rendon N.M., St John E.A., Demas G.E. (2017). Sex-specific modulation of the gut microbiome and behavior in Siberian hamsters. Brain Behav. Immun..

[51] Jasnow A.M., Huhman K.L., Bartness T.J., Demas G.E. (2000). Short-day increases in aggression are inversely related to circulating testosterone concentrations in male Siberian hamsters (*Phodopus sungorus*). Horm. Behav..

[52] Rendon N.M., Rudolph L.M., Sengelaub D.R., Demas G.E. (2015). The agonistic adrenal: Melatonin elicits female aggression via regulation of adrenal androgens. Proc. Biol. Sci..

[53] Rendon N.M., Soini H.A., Scotti M.A., Weigel E.R., Novotny M.V., Demas G.E. (2016). Photoperiod and aggression induce changes in ventral gland compounds exclusively in male Siberian hamsters. Horm. Behav..

[54] Ren C.C., Sylvia K.E., Munley K.M., Deyoe J.E., Henderson S.G., Vu M.P., Demas G.E. (2020). Photoperiod modulates the gut microbiome and aggressive behavior in Siberian hamsters. J. Exp. Biol..

[55] Cusick J.A., Wellman C.L., Demas G.E. (2022). Maternal stress and the maternal microbiome have sex-specific effects on offspring development and aggressive behavior in Siberian hamsters (*Phodopus sungorus*). Horm. Behav..

[56] Shor E.K., Brown S.P., Freeman D.A. (2022). Bacteria and bellicosity: Photoperiodic shifts in gut microbiota drive seasonal aggressive behavior in male Siberian hamsters. J. Biol. Rhythm..

[57] Albers H.E., Huhman K.L., Meisel R.L., Pfaff D.W., Arnold A.P., Fahrbach S.E., Etgen A.M., Rubin R.T. (2002). Hormonal basis of social conflict and communication. Hormones, Brain and Behavior.

[58] Jia Y., Jin S., Hu K., Geng L., Han C., Kang R., Pang Y., Ling E., Tan E.K., Pan Y. (2021). Gut microbiome modulates Drosophila aggression through octopamine signaling. Nat. Commun..

[59] Grinberg M., Levin R., Neuman H., Ziv O., Turjeman S., Gamliel G., Nosenko R., Koren O. (2022). Antibiotics increase aggression behavior and aggression-related pheromones and receptors in *Drosophila melanogaster*. iScience.

[60] Wang L., Anderson D.J. (2010). Identification of an aggression-promoting pheromone and its receptor neurons in *Drosophila*. Nature.

[61] Schlüter T., Winz O., Henkel K., Prinz S., Rademacher L., Schmaljohann J., Dautzenberg K., Cumming P., Kumakura Y., Rex S. (2013). The impact of dopamine on aggression: An [^18^F]-FDOPA PET Study in healthy males. J. Neurosci..

[62] Langmajerová M., Roubalová R., Šebela A., Vevera J. (2023). The effect of microbiome composition on impulsive and violent behavior: A systematic review. Behav. Brain Res..

[63] Tcherni-Buzzeo M. (2023). Dietary interventions, the gut microbiome, and aggressive behavior: Review of research evidence and potential next steps. Aggress. Behav..

[64] Hill C., Guarner F., Reid G., Gibson G.R., Merenstein D.J., Pot B., Morelli L., Canani R.B., Flint H.J., Salminen S. (2014). The International Scientific Association for probiotics and prebiotics consensus statement on the scope and appropriate use of the term probiotic. Nat. Rev. Gastroenterol. Hepatol..

[65] Gibson G.R., Hutkins R., Sanders M.E., Prescott S.L., Reimer R.A., Salminen S.J., Scott K., Stanton C., Swanson K.S., Cani P.D. (2017). Expert consensus document: The International Scientific Association for Probiotics and Prebiotics (ISAPP) consensus statement on the definition and scope of prebiotics. Nat. Rev. Gastroenterol. Hepatol..

[66] Roberfroid M., Gibson G.R., Hoyles L., McCartney A.L., Rastall R., Rowland I., Wolvers D., Watzl B., Szajewska H., Stahl B. (2010). Prebiotic effects: Metabolic and health benefits. Br. J. Nutr..

[67] Pluznick J.L. (2016). Gut microbiota in renal physiology: Focus on short-chain fatty acids and their receptors. Kidney Int..

[68] Canani R.B., Costanzo M.D., Leone L., Pedata M., Meli R., Calignano A. (2011). Potential beneficial effects of butyrate in intestinal and extraintestinal diseases. World J. Gastroenterol..

[69] Rivière A., Gagnon M., Weckx S., Roy D., De Vuyst L. (2015). Mutual cross-feeding interactions between *Bifidobacterium longum* subsp. longum NCC2705 and Eubacterium rectale ATCC 33656 explain the bifidogenic and butyrogenic effects of arabinoxylan oligosaccharides. Appl. Environ. Microbiol..

[70] Boets E., Gomand S.V., Deroover L., Preston T., Vermeulen K., De Preter V., Hamer H.M., Van den Mooter G., De Vuyst L., Courtin C.M. (2017). Systemic availability and metabolism of colonic-derived short-chain fatty acids in healthy subjects: A stable isotope study. J. Physiol..

[71] Burokas A., Arboleya S., Moloney R.D., Peterson V.L., Murphy K., Clarke G., Stanton C., Dinan T.G., Cryan J.F. (2017). Targeting the Microbiota-Gut-Brain Axis: Prebiotics Have Anxiolytic and Antidepressant-like Effects and Reverse the Impact of Chronic Stress in Mice. Biol. Psychiatry.

[72] American Psychiatric Association (2013). Diagnostic and Statistical Manual of Mental Disorders.

[73] Fung L.K., Mahajan R., Nozzolillo A., Bernal P., Krasner A., Jo B., Coury D., Whitaker A., Veenstra-Vanderweele J., Hardan A.Y. (2016). Pharmacologic treatment of severe irritability and problem behaviors in autism: A systematic review and meta-analysis. Pediatrics.

[74] Lecavalier L. (2006). Behavioral and emotional problems in young people with pervasive developmental disorders: Relative prevalence, effects of subject characteristics, and empirical classification. J. Autism Dev. Disord..

[75] Nikolov R.N., Bearss K.E., Lettinga J., Erickson C., Rodowski M., Aman M.G., McCracken J.T., McDougle C.J., Tierney E., Vitiello B. (2009). Gastrointestinal symptoms in a sample of children with pervasive developmental disorders. J. Autism Dev. Disord..

[76] Tan Q., Orsso C.E., Deehan E.C., Kung J.Y., Tun H.M., Wine E., Madsen K.L., Zwaigenbaum L., Haqq A.M. (2021). Probiotics, prebiotics, Synbiotics, and fecal microbiota transplantation in the treatment of behavioral symptoms of autism spectrum disorder: A systematic review. Autism Res..

[77] Aman M.G., Singh N.N., Stewart A.W., Field C.J. (1985). The aberrant behavior checklist: A behavior rating scale for the assessment of treatment effects. Am. J. Ment. Defic..

[78] Shaaban S.Y., El Gendy Y.G., Mehanna N.S., El-Senousy W.M., El-Feki H.S.A., Saad K., El-Asheer O.M. (2018). The role of probiotics in children with autism spectrum disorder: A prospective, open-label study. Nutr. Neurosci..

[79] Arnold L.E., Luna R.A., Williams K., Chan J., Parker R.A., Wu Q., Hollway J.A., Jeffs A., Lu F., Coury D.L. (2019). Probiotics for gastrointestinal symptoms and quality of life in autism: A placebo-controlled pilot trial. J. Child Adolesc. Psychopharmacol..

[80] Liu Y.W., Liong M.T., Chung Y.E., Huang H.Y., Peng W.S., Cheng Y.F., Lin Y.S., Wu Y.Y., Tsai Y.C. (2019). Effects of *Lactobacillus plantarum* PS128 on children with autism spectrum disorder in Taiwan: A randomized, double-blind, placebo-controlled trial. Nutrients.

[81] Niu M., Li Q., Zhang J., Wen F., Dang W., Duan G., Li H., Ruan W., Yang P., Guan C. (2019). Characterization of intestinal microbiota and probiotics treatment in children with autism spectrum disorders in China. Front. Neurol..

[82] Santocchi E., Guiducci L., Prosperi M., Calderoni S., Gaggini M., Apicella F., Tancredi R., Billeci L., Mastromarino P., Grossi E. (2020). Effects of probiotic supplementation on gastrointestinal, sensory and core symptoms in autism spectrum disorders: A randomized controlled trial. Front. Psychiatry.

[83] Sanctuary M.R., Kain J.N., Chen S.Y., Kalanetra K., Lemay D.G., Rose D.R., Yang H.T., Tancredi D.J., German J.B., Slupsky C.M. (2019). Pilot study of probiotic/colostrum supplementation on gut function in children with autism and gastrointestinal symptoms. PLoS ONE.

[84] Van Nood E., Vrieze A., Nieuwdorp M., Fuentes S., Zoetendal E.G., de Vos W.M., Visser C.E., Kuijper E.J., Bartelsman J.F., Tijssen J.G. (2013). Duodenal infusion of donor feces for recurrent Clostridium difficile. N. Engl. J. Med..

[85] Kang D.W., Adams J.B., Gregory A.C., Borody T., Chittick L., Fasano A., Khoruts A., Geis E., Maldonado J., McDonough-Means S. (2017). Microbiota Transfer Therapy alters gut ecosystem and improves gastrointestinal and autism symptoms: An open-label study. Microbiome.

[86] Kang D.W., Adams J.B., Coleman D.M., Pollard E.L., Maldonado J., McDonough-Means S., Caporaso J.G., Krajmalnik-Brown R. (2019). Long-term benefit of microbiota Transfer Therapy on autism symptoms and gut microbiota. Sci. Rep..

[87] Vuong H.E., Pronovost G.N., Williams D.W., Coley E.J.L., Siegler E.L., Qiu A., Kazantsev M., Wilson C.J., Rendon T., Hsiao E.Y. (2020). The maternal microbiome modulates fetal neurodevelopment in mice. Nature.

[88] Mikami K., Takahashi H., Kimura M., Isozaki M., Izuchi K., Shibata R., Sudo N., Matsumoto H., Koga Y. (2009). Influence of maternal bifidobacteria on the establishment of bifidobacteria colonizing the gut in infants. Pediatr. Res..

[89] Sirilun S., Takahashi H., Boonyaritichaikij S., Chaiyasut C., Lertruangpanya P., Koga Y., Mikami K. (2015). Impact of maternal bifidobacteria and the mode of delivery on *Bifidobacterium* microbiota in infants. Benef. Microbes.

[90] Takahashi H., Mikami K., Nishino R., Matsuoka T., Kimura M., Koga Y. (2010). Comparative analysis of the properties of bifidobacterial isolates from fecal samples of mother–infant pairs. J. Pediatr. Gastroenterol. Nutr..

[91] Makino H., Kushiro A., Ishikawa E., Muylaert D., Kubota H., Sakai T., Oishi K., Martin R., Ben Amor K., Oozeer R. (2011). Transmission of intestinal *Bifidobacterium longum* subsp. longum strains from mother to infant, determined by multilocus sequencing typing and amplified fragment length polymorphism. Appl. Environ. Microbiol..

[92] Makino H., Kushiro A., Ishikawa E., Kubota H., Gawad A., Sakai T., Oishi K., Martin R., Ben-Amor K., Knol J. (2013). Mother-to-infant transmission of intestinal bifidobacterial strains has an impact on the early development of vaginally delivered infant’s microbiota. PLoS ONE.

[93] Makino H. (2018). Bifidobacterial strains in the intestines of newborns originate from their mothers. Biosci. Microbiota Food Health.

